# Biological Bases of Empathy and Social Cognition in Patients with Attention-Deficit/Hyperactivity Disorder: A Focus on Treatment with Psychostimulants

**DOI:** 10.3390/brainsci11111399

**Published:** 2021-10-24

**Authors:** Pamela Fantozzi, Gianluca Sesso, Pietro Muratori, Annarita Milone, Gabriele Masi

**Affiliations:** 1 IRCCS Stella Maris Foundation, Scientific Institute of Child Neurology and Psychiatry, Department of Child and Adolescent Psychiatry and Psychopharmacology, 56128 Pisa, Italy; pamela.fantozzi@fsm.unipi.it (P.F.); gianluca.sesso@fsm.unipi.it (G.S.); pietro.muratori@fsm.unipi.it (P.M.); annarita.milone@fsm.unipi.it (A.M.); 2 Department of Clinical and Experimental Medicine, University of Pisa, 56126 Pisa, Italy

**Keywords:** empathy, theory of mind, emotion recognition, ADHD, disruptive behavior

## Abstract

In recent years, there has been growing interest in investigating the effect of specific pharmacological treatments for ADHD not only on its core symptoms, but also on social skills in youths. This stands especially true for ADHD patients displaying impulsive aggressiveness and antisocial behaviors, being the comorbidity with Disruptive Behavior Disorders, one of the most frequently observed in clinical settings. This systematic review aimed to synthesize research findings on this topic following PRISMA guidelines and to identify gaps in current knowledge, future directions, and treatment implications. Search strategies included the following terms: ADHD; methylphenidate and other ADHD drugs; empathy, theory of mind and emotion recognition. Full-text articles were retrieved and data from individual studies were collected. Thirteen studies were finally included in our systematic review. Ten studies assessing changes in empathy and/or theory of mind in patients with ADHD treated after pharmacological interventions were identified. Similarly, seven partially overlapping studies assessing changes in emotion recognition were retrieved. Despite a great heterogeneity in the methodological characteristics of the included studies, most of them reported an improvement in emphatic and theory of mind abilities in youths with ADHD treated with psychostimulants and nonstimulant drugs, as well as positive but less consistent results about emotion recognition performances.

## 1. Introduction

Attention-Deficit/Hyperactivity Disorder (ADHD) is one of the most common neurodevelopmental disorders, with a pooled worldwide prevalence of 7.2% among children and adolescents [1]. In addition to the core symptoms of inattention, hyperactivity, and impulsivity [2], subjects with ADHD frequently exhibit difficulties in establishing and keeping relationships with peers and are perceived as less socially competent than peers [3]. Particularly, they tend to show a high rate of social and interpersonal problems during their whole life span, since reduced social cognition skills are usually found to be highly associated with the disorder, which may be considered to be an independent risk factor for negative outcome and poor quality of life in ADHD [3].

Interestingly, ADHD core symptoms per se may interfere with adequate social interactions. Indeed, attention deficits interfere with a proper coding and interpretation of social information, i.e., focusing and sustaining attention during conversations or appropriately reading social cues during play [4]. On the other hand, impulsivity involves inappropriately intruding in conversations or play, and disinhibition of motor, verbal, and behavioral responses can lead to fewer opportunities for social interaction due to peer rejection [5]. Moreover, ADHD is commonly associated with the presence of comorbid disruptive behavior disorders such as Oppositional Defiant Disorders (ODD) and/or Conduct Disorders (CD) that may further worsen social impairments and maladjustment [6]. Interestingly, deficits in social cognition skills may be even more challenging when ADHD presents with comorbid ODD/CD [7].

### 1.1. Empathy and Related Constructs

Social cognition is essential for successful social interaction and, as a whole, refers to the ability to understand other people’s behaviors. It involves codification, representation and interpretation of social cues and includes (1) recognizing others’ affects from face and prosody perception (i.e., emotion recognition), (2) making inferences regarding others’ mental states (i.e., theory of mind (ToM)), (3) sharing and understanding the emotional perspective of others (i.e., empathy) [3]. More complex social cognition abilities include humor processing and further steps of the social information-processing model [8], from which biases may lead individuals to assume the hostile attributions of another’s ambiguous behavior and generate aggressive or ineffective solutions to social problems.

Empathy is a complex multidimensional construct including an affective component—affective empathy (AE)—that is, the capacity of sharing emotions and responding to emotional displays of others, and a cognitive one—cognitive empathy (CE)—namely, the ability to understand the perspective of another person [9,10,11,12,13]. AE may involve several related underlying processes, including, among others, emotional contagion, emotion recognition, and shared pain [14]; on the other hand, CE involves making inferences regarding the other’s affective and cognitive mental states [15].

These two components may have different neuroanatomical correlates, the former implying the contribution of limbic and paralimbic structures and developing earlier than the latter, which assumes, in turn, a fine-tuned maturation of prefrontal and temporal networks [16]. However, in a study on the anatomical correlates of empathy in patients with focal brain injuries, Shamay-Tsoory and colleagues [17] demonstrated that prefrontal lesions, especially those involving the orbitofrontal and ventromedial regions, were significantly associated with impairments in both cognitive and affective empathic skills, while lesions involving right parietal areas were also associated with deficient empathy. The distinction between the emotional and cognitive empathic subprocesses may also relate to different neurobiological systems. It has been suggested, indeed, that the oxytocinergic system, which has been associated with attachment and pair bonding, may modulate emotional but not cognitive empathy [18], whereas dopaminergic circuitry is associated with cognitive aspects of empathy [19].

Although the two systems work independently, as previously suggested by the Perception–Action Model of empathy [20,21], they interact with each other. The affective component is, indeed, regarded as a bottom-up automatic process, while the cognitive component may be better represented as a top-down modulator. Nonetheless, they also work in synergy with several other distinct but integrated components of social cognition, among which the theory of mind (ToM) stands out first. ToM is defined as the ability to make inferences regarding others’ mental states—their knowledge, needs, intentions, and feelings—and is mediated by dissociable though interacting cognitive and affective aspects [22], whereas cognitive ToM, for instance, assessed through the so-called False Belief task, is thought to require cognitive understanding of the difference between the speaker’s knowledge and that of the listener. Affective ToM, for example, tested with Faux Pas and Irony tasks, is supposed to require, in addition, an empathic appreciation of the listener’s emotional state [23]. ToM functioning critically involves a complex neural network including the medial prefrontal cortex, the superior temporal sulcus region, the temporal pole, and the amygdalae [24,25,26], and has also been linked to the integrity of the dopaminergic and serotoninergic systems [24].

On the other side, the appropriate recognition of emotional cues represents a fundamental milestone in the early development of social cognition skills. Indeed, the ability to identify emotions from facial expressions and prosody is acquired during childhood and further develops during adolescence. Besides, nonverbal channels of communication seem to play an important role in helping individuals to interact appropriately with each other. Intact emotion recognition is required for the inhibition of aggressive behavior and its deficiencies might lead to aggressive reactions toward others [27]. At the same time, impaired recognition of facial emotions has been suggested to play a central role for social malfunctioning, being a potential cause of low social competence and low popularity in peer groups [28]. In other words, social adaptation is poorer in those who tend to identify emotional expressions less accurately [29].

In healthy individuals, facial expressions usually elicit neural changes over frontotemporal and parieto-occipital cortices, while right-sided peri-sylvian areas are engaged in the processing of emotional prosody [3]. Face emotion recognition has also been linked to temporal, prefrontal (e.g., orbitofrontal), and anterior cingulate regions, as well as the amygdala and the basal ganglia [30]. Finally, a connection between the perception of emotions and the dopaminergic pathway has been demonstrated [31].

### 1.2. Social Cognition in ADHD

Clinical evidence suggests several psychopathological disorders to be characterized by deficits in social cognition [32,33]. Importantly, empathy/ToM deficits have been implicated, indeed, in neurodevelopmental conditions in childhood and adolescence, among which Autism Spectrum Disorder (ASD) [34,35,36,37] and ODD/CD with limited prosocial emotions i.e., callous–unemotional traits [38,39] are the most studied. Empathy and ToM may be also compromised in a proportion of youths with ADHD. Clinical practice usually reveals low levels of social perspective taking and ToM in ADHD children [6,40,41]. Indeed, young people with ADHD may have low CE attitudes, as demonstrated, for instance, by the frequently observed unawareness of other children playing the same game [42]. In this regard, a recent meta-analysis on social cognition findings in ADHD [43] revealed that especially ToM was significantly impaired in ADHD patients. Interestingly, they also reported that social cognition deficits in ADHD lied intermediately between ASD and healthy controls [43].

On the other hand, several studies have also demonstrated that children with ADHD exhibited AE deficits compared to healthy controls [6,41,44], either assessed as trait using parent reports [45] or as a state assessed with affective responses to vignettes [41]. Presumably, a global empathy deficit can be detected in ADHD, involving both components, as shown by Maoz and colleagues [46] through self-report assessments. Interestingly, in another study from the same research group [47], differences in the empathic profile were identified between the Combined (ADHD-C) and the Inattentive (ADHD-I) subtypes of ADHD, with greater impairment in the former.

Children and adolescents with ADHD also exhibit an impaired emotion recognition ability and a nonverbal receptive language deficit [5,48,49], which denotes a difficulty in detecting and interpreting social clues and generates impaired social interactions and interpersonal problems. In particular, individuals with ADHD are significantly poorer in identifying emotional expressions, especially the negative effects of fear, anger and sadness, likely originating from a primary deficiency in encoding social cues and selectively inhibiting irrelevant information in ADHD [43,50].

According to Uekermann and colleagues [3], empathy deficits in ADHD might be explained, at least in part, by the impulsive response modalities typically found in these patients, and thus may be linked to dysfunctions of the fronto-striatal brain networks, functionally related to empathic processing and executive functioning. Interestingly, Barkley [51] argues that deficits in behavioral inhibition might impair social cognition skills, but how much they could affect empathic abilities still remains an unsolved question. In this respect, several studies have demonstrated a significant positive correlation between empathic skills and executive functions in both healthy subjects [52] and clinical samples [7,53,54]. Interestingly, a recent meta-analysis on healthy individuals [52] revealed that executive functioning, i.e., working memory, cognitive flexibility, and sustained attention, was more strongly related to CE; besides, AE was still closely related to inhibitory control. Conversely, Cristofani and colleagues [7] identified an opposite trend in ADHD patients and speculated that these subjects are somewhat constrained by their executive dysfunction in an underdeveloped empathic attitude, which would be limited to the expression of emotional contagion.

### 1.3. The Systematic Review

Recent literature has suggested that pharmacological interventions in ADHD patients may provide beneficial effects on social cognition deficits. Indeed, psychostimulants, including methylphenidate (MPH), and amphetamines, the gold-standard drug treatment for ADHD [55], have been likely associated with improvements in social judgment and interpersonal relationships [56,57], as well as in empathy and ToM in youths with ADHD [46,47,58,59,60,61,62]. Interestingly, MPH administration has been shown to promote empathy-like behaviors and sociability and reduce aggressiveness in a mouse model of callousness [63]. Moreover, it has been suggested that MPH treatment may possibly provide an improvement in emotion recognition [28,50]. Nonetheless, the evidence on the efficacy of psychostimulants on empathy and ToM, as well as on emotion recognition, is still under debate [64]. Thus, the aim of the present study was to systematically review the available literature on the topic in order to clarify whether the gold-standard drug treatment for ADHD may exert its effects on empathy and related constructs, through and beyond its well-known effects on the core symptoms of the disorder.

## 2. Materials and Methods

### 2.1. Search Strategy

The aim of the present study was to perform a systematic review of the literature on the effects of psychostimulants and nonstimulant drugs on empathy and related constructs in patients with ADHD. The review was conducted according to the Preferred Reporting Items for Systematic Reviews and Meta-Analyses (PRISMA) guidelines; the corresponding checklist is available in Supplementary Table S1. The protocol of the present systematic review was preregistered on PROSPERO (CRD42021247024). Three bibliographic databases were searched, namely PubMed, Scopus, and Web of Science, from the inception date to the 10 August 2021. A search strategy was developed including three groups of terms related to the following semantic fields: (1) ADHD; (2) Methylphenidate or other psychostimulants and nonstimulant drugs for ADHD; (3) Social Cognition, Empathy, Theory of Mind and Emotion Recognition. The full search strategy, along with the details of the bibliographic search, is available in Supplementary Table S2. The strategy was thus to include all relevant articles relating to Group 1 and Group 2 and Group 3; terms were consistently adapted for each database. Results of the bibliographic search were then downloaded into Mendeley software, and two authors (GS and PF) reviewed and discussed the scoping search which included both original studies and reviews. If a previous review was already available on the topic, its reference list was carefully searched to retrieve primary studies. Reference lists of the studies included in the final search were also thoroughly inspected to identify relevant citations.

### 2.2. Screening Procedure

**Figure 1:**
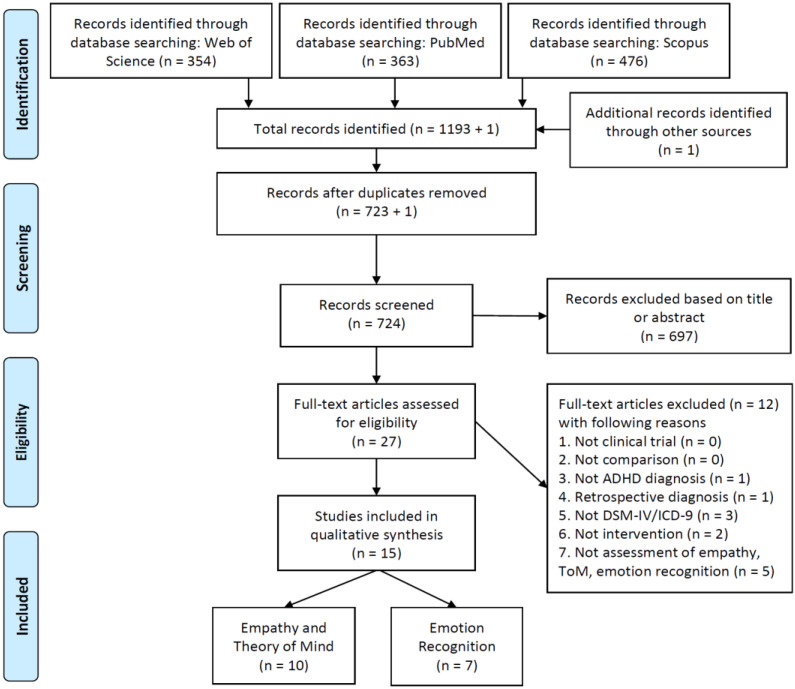
PRISMA flowchart showing the process of identification and selection of studies.

Our search strategy was used to retrieve potentially relevant abstracts; duplicates from different bibliographic databases were initially removed, whereas additional records were identified through reference lists and the inspection of screened articles, as stated above, were also included. Two researchers (GS and PF) screened all titles and abstracts to identify relevant articles. Full texts of selected papers were then retrieved and carefully screened to finally identify the included studies according to eligibility criteria. Any disagreements were resolved by consensus. The PRISMA flowchart (Figure 1) shows the process of identification and selection of papers. Inclusion criteria were defined in order to retrieve clinical studies aimed at assessing the effects of MPH and other psychostimulants and nonstimulant drugs on empathy, theory of mind, and emotion recognition in patients with ADHD, as follows:

(1) Study design: any type of clinical trial;
(2) Comparison: either case versus control, drug versus placebo or pre-to peri-/post-treatment;
(3) Participants: patients non-retrospectively diagnosed with ADHD according to the international classification systems DSM-IV, ICD-9, or later versions; no restriction for participants’ age, gender, or IQ;
(4) Intervention: either one-day, single-dose administration or prolonged daily administration of psychostimulants (e.g., Methylphenidate) or nonstimulant drugs (e.g., Atomoxetine);
(5) Measures: any type of measurement (i.e., tasks, rating scales, and parent- or self-rated questionnaires) assessing empathy, theory of mind, and emotion recognition.

Exclusion criteria are detailed in Figure 1 and Supplementary Table S2. Briefly, in order of exclusion, they have been defined as follows: (1) Not clinical trial; (2) Absence of adequate comparison; (3) Subjects not diagnosed with ADHD; (4) Retrospective diagnosis of ADHD; (5) Clinical diagnosis not based on DSM-IV, ICD-9, or later versions; (6) Not intervention with psychostimulant/nonstimulant drugs; (7) Not assessment of empathy, theory of mind, and emotion recognition.

### 2.3. Data Collection

For each included study, we extracted relevant information, whenever available, including sample size, demographic data (age and gender), ADHD diagnosis and subtypes, intellectual functioning and psychiatric comorbidities, previous and current medication (including dosage and administration), follow-up duration, as well as information about the clinical measure used to assess changes in empathic competencies and related constructs and main findings of the study. When datasets were not fully available, authors of the included studies were contacted to attain the relevant data. Extracted information are available in Table 1 and Table 2. Included studies were classified according to the examined construct as follows: (1) empathy and theory of mind and (2) emotion recognition. Emotion recognition is an underlying process related to the affective empathy, often investigated separately from the construct of empathy and theory of mind; for this reason, we decided to group studies into two classification types. Articles were also grouped according to the study design, either (1) single-dose administration of the drug with one-day follow-up or (2) daily administration of the medication with prolonged follow-up.

## 3. Results

Details of the screening process and the identification and selection of papers are available in Figure 1, along with the main reasons for exclusion. In summary, 1193 abstracts were initially retrieved using our search strategy, plus one additional record identified in the reference lists of the studies included in the final search. After duplicates removal, 724 records were screened by two authors (G.S. and P.F.) and any disagreement was resolved by consensus. Twenty-seven full-text articles were carefully assessed for eligibility, of which 12 were excluded. Fifteen articles were finally included in our systematic review and were non-mutually subdivided into two partially overlapping groups as follows: (1) empathy and theory of mind (n = 10 studies); (2) emotion recognition (n = 7 studies).

### 3.1. Empathy and Theory of Mind

Ten studies [46,47,58,59,60,61,62,65,66,67] assessing the effects of MPH (either immediate-release or long-acting formulations) on empathy and ToM in young patients with ADHD were finally identified. One study [58] also assessed the effects of atomoxetine (ATX) treatment, while another one [65] compared unimodal (medication only) versus multimodal (medication plus cognitive behavioral therapy).

All studies were conducted on children and adolescents aged 6 to 18 years old. Diagnoses were based on DSM-IV or DSM-5 systems [2] and included different proportions of ADHD subtypes. Intelligence was on average, while other psychiatric comorbidities were excluded based on standardized criteria, except for the study by Fantozzi and colleagues [62], which also included patients with ADHD and comorbid with language disorders, verbal dyspraxia, Specific Learning Disabilities, tics, affective disorders, and behavioral disruptive disorders, and for the study by Golubchik and Weizman [59], which also included youths with ADHD and comorbid with ODD. Further details of the studies are reported in Table 1.

Five studies [46,47,61,66,67] examined the effect of a single-dose administration of MPH on children and adolescents with ADHD who were already regularly taking the medication at the time of the study. Particularly, one study [47] revealed an improvement in cognitive and affective ToM, as measured with two ToM tests, the Faux Pas Recognition task and the ToM Computerized task, in a group of young patients with ADHD after a single MPH dose administration. The same research group [46] later replicated their findings through a self-report measure, the Interpersonal Reactivity Index, and the Faux Pas test demonstrating that ADHD patients, who initially displayed significant deficits in empathy/ToM skills, improved their performances after a single dose of MPH until they matched their healthy peers.

A recent study [61] corroborated these findings by examining the effect of a single dose of MPH versus placebo on different ToM task performances in a group of children with ADHD versus healthy controls in a double-blind controlled trial. ToM abilities in ADHD children, while initially poorer, normalized only after MPH administration and differences between the two groups were no longer found. The same research group, analyzing later the same ADHD sample [67], found a correlation between the severity of the ADHD behavioral symptoms and deficits in ToM. The authors also found that the administration of a single dose of MPH improved ToM performance, especially in children with more severe behavioral symptoms. Conversely, another research group [66] revealed no significant single-dose MPH effects in children with ADHD on ToM performances, as measured through the commonly used Reading the Mind in the Eyes test.

Five additional studies [58,59,60,62,65] evaluated the effects of mid-term treatment with daily drug administrations in ADHD patients. Notably, all five studies agreed in demonstrating a significant improvement in empathy/ToM performance after drug treatment. Particularly, a significant increase in empathic abilities, as measured by the Empathizing Quotient, was shown after 12 weeks of daily treatment with MPH [59]; it should be noted, however, that half of the included patients were also diagnosed with comorbid ODD, which implied even lower baseline scores than the ADHD-only group. Though they did not confirm such improvement in trait empathy by means of two paper-and-pencil questionnaires, the Bryant Index of Empathy and the Griffith Empathy Measure-Parent Rating, Gumustas and colleagues [60] found a significant increase in state empathic skills, as measured through the Empathy Response Task, after 12 weeks of MPH treatment in drug-naïve children and adolescents with ADHD. Recently, our research group [62] conducted a study on a sample of drug-naïve young patients with ADHD, naturalistically treated with MPH monotherapy and followed up for 6 months, who showed a significant improvement in AE and CE scores measured with the Basic Empathy Scale. The authors also found that changes in attention symptoms predicted changes in AE but not in CE.

Interestingly, a multimodal approach including drug treatment plus cognitive behavior therapy resulted in significantly greater improvements in frequency indicators on skillful reactions of empathy than the medication-only approach in ADHD patients [65]. Finally, ATX demonstrated a similar effectiveness on ToM skills, as measured with the Reading the Mind in the Eyes test, in a group of young drug-naive patients with ADHD, as compared with long-acting MPH administered for 12 weeks [58].

### 3.2. Emotion Recognition

Seven studies [31,50,58,60,68,69,70] assessing the effects of psychostimulants and nonstimulant drugs on emotion recognition abilities in individuals with ADHD were finally identified. Most of the studies were conducted on children and adolescents aged 7 to 17 years old, except for one study [69] that included adult patients. Diagnoses were based on DSM-IV and, in one case [31], on the ICD classification system, and included different proportions of ADHD subtypes; intelligence was generally on average, whereas other psychiatric comorbidities were excluded based on standardized criteria except for two studies [50,68] that included learning disabilities and internalizing disorders, respectively. Further details of the studies are reported in Table 2.

Two studies [31,68] examined the effect of a single-dose administration of MPH on children with ADHD who were already taking the medication regularly at the time of the study. Particularly, the former study [68] compared 30 ADHD patients with and without learning disabilities (LD) to 15 matched controls with no ADHD nor LD and found that, while at baseline only ADHD patients with comorbid LD demonstrated greater difficulties in perceiving paralanguage gesture cues than the other groups, as assessed through the Diagnostic Analysis of Nonverbal Accuracy test, the effect of medication was to equalize such differences. On the other hand, the latter study [31] revealed no significant medication effects on 21 children with ADHD on facial affect recognition abilities, neither with pictures of faces nor with eye pairs for any type of emotions, as assessed through the Frankfurt Test and Training of Social Affect.

Four studies [50,58,60,70] evaluated the effect of a mid-term treatment with daily administration of MPH. The oldest study [50] revealed significant improvements in fear and anger recognition in thirty-three patients with ADHD and comorbid anxiety and depression symptoms—either drug-naïve or under MPH treatment suspended at least three days before the testing session—after four weeks of daily treatment with MPH. Nonetheless, despite such improvements, ADHD patients still displayed deficits in emotion recognition abilities compared to healthy controls. Conversely, in a cross-sectional design study [70], the authors found no significant effects of MPH on emotion recognition reaction times and the number of correct answers in twenty-eight treated versus twenty-eight untreated ADHD patients compared to healthy controls, by means of a morphing task implemented from the Karolinska Directed Emotional Faces Set. The only difference approaching statistical significance concerned the number of sad faces mistaken as angry after MPH treatment.

Two more recent studies [58,60] compared a sample of more than 60 drug-naïve patients treated with MPH for 3 months to matched healthy controls and found significant improvements in emotion recognition abilities, as assessed through the Diagnostic Analysis of Nonverbal Accuracy (for sadness and anger only) and the Benton Face Recognition tests, respectively. Interestingly, the former study [58] confirmed a similar trend also for ATX. Finally, only one study [69] assessed the effects of a 4-week treatment with lisdexamfetamine (LDX) versus placebo in 25 adult patients with ADHD on performances of a mixed task evaluating executive functioning and emotion recognition abilities (i.e., Face Emotion Go/No-Go Task). The authors revealed no significant differences between the two treatment arms; it should be noted, however, that some patients were previously treated with MPH suspended at least 2 weeks before the testing session.

## 4. Discussion

The present systematic review aimed to synthesize research findings on the effect of psychostimulants and nonstimulant drugs on social cognition in patients with ADHD. As far as we know, our study is the first review that systematically and specifically addressed this topic; former narrative but still comprehensive reviews [3,6] were respectively focused on social dysfunctions in ADHD, with the contribution of comorbid disruptive behavior disorders (i.e., ODD/CD) to social impairments, and on the link between social cognition deficits in ADHD and evidence from neuroimaging and lesion studies. Here, we complementarily aimed at looking for the available evidence from scientific literature on the impact of pharmacological interventions on empathy, theory of mind, and emotion recognition in ADHD. The research interest on such a topic is quite recent since the oldest studies retrieved through our search date back to 1999 for emotion recognition [68] and even later for empathy/ToM [47]. Unfortunately, for this reason, the number of studies dedicated to the assessment of social cognition in ADHD is still limited and even less on the effects of pharmacological treatment.

Most of the studies we identified through our search were conducted on children as expected, since ADHD is a neurodevelopmental condition with greater incidence in childhood [71] and social cognition deficits may become attenuated from adolescence on [53], while only one study was performed on adults [69]. Nonetheless, longitudinal studies are missing to investigate the developmental trajectories of social cognition skills from early childhood to adulthood, thus highlighting a still underexplored field of research on this topic. Study samples included, when clinical details were available, both Combined and Inattentive presentations of ADHD, whereas the Hyperactive/Impulsive type was typically underrepresented; in one study [68], a group of patients with ADHD and comorbid learning disabilities was compared to a pure ADHD group, while DBD and other comorbidities were generally excluded with some notable exceptions [31,50,59,62]. Most studies also included a comparison group variably consisting of healthy or clinical controls without ADHD.

As for the assessment of empathy and ToM, both paper-and-pencil questionnaires, including self (e.g., Basic Empathy Scale) and parent reports (e.g., Griffith Empathy Measurement), and standardized tests (e.g., Reading the Mind in the Eyes Test) were used. On the other hand, only standardized tasks were used instead to assess the emotion recognition abilities of participants (e.g., Diagnostic Analysis of Nonverbal Accuracy). Among the available treatment options for ADHD, most studies assessed the effect of psychostimulants, and first of all MPH, which represents the gold-standard pharmacological intervention for the disorder [55]. Schulz and colleagues [69], instead, evaluated the impact of LDX—an amphetamine derivative—while the study conducted by Demirci and Erdogan [58] was the only one that applied a selective noradrenaline reuptake inhibitor, namely ATX. Finally, Coelho and colleagues [65] assessed the efficacy of a multimodal treatment (medication plus cognitive-behavioral therapy) to investigate a possible additive effect of both types of intervention on social skills.

Study designs significantly varied across the included papers, most of which were based on longitudinal trials. Indeed, several studies assessed the effect of a mid-term treatment with MPH and/or other pharmacological and non-pharmacological interventions administered on a regular daily basis for 3–12 weeks to 4–6 months, typically in drug-naïve ADHD patients, except for a few samples including individuals previously exposed to psychostimulants that were washed-out from 3 days to 1 month prior to testing. Some additional studies assessed the effects of a single-dose administration of MPH, either alone or versus placebo in drug-naïve ADHD patients or subjects that were already regularly taking drugs, whereas only one study [70] applied a cross-sectional design on drug-naïve ADHD patients versus MPH-treated ones.

Eight studies demonstrated significant improvements in empathy and/or ToM skills after a single-dose administration or prolonged treatment [46,47,58,59,60,61,62]. Interestingly, Coelho and colleagues [65] observed a significant effect of a multimodal treatment including medication combined with CBT on measures of social skills. Notably, the only study in which the authors did not find any significant improvement of ToM skills after a single-dose administration of MPH was the one by Golubchik and Weizman [66]. When compared to healthy controls, patients with ADHD displayed significantly lower baseline performances on empathy/ToM measures that greatly improved after pharmacological intervention until they reached those obtained by the comparison group [46,58,61]; interestingly, the greater baseline impairments were identified in Hyperactive/Impulsive and Combined types than in the Inattentive one.

As for emotion recognition abilities, most studies showed a significant improvement after the implementation of pharmacological treatment [50,58,60,68], while one study [31] found a non-significant improvement. Only two studies did not reveal a beneficial effect, though neither was it detrimental, of drugs on the emotion recognition skills in ADHD patients [69,70]; however, the former assessed the effects of LDX, which is not considered the first-line treatment option for the disorder, while the latter was based on a cross-sectional design. Interestingly, Gumustas and colleagues [60] revealed that, following the treatment with MPH, the ADHD group showed a significant improvement in the recognition of anger and sadness expressions. When compared to controls, patients with ADHD exhibited significantly lower baseline performances on emotion recognition that improved after pharmacological implementation until they reached those obtained by the comparison group.

**Figure 2:**
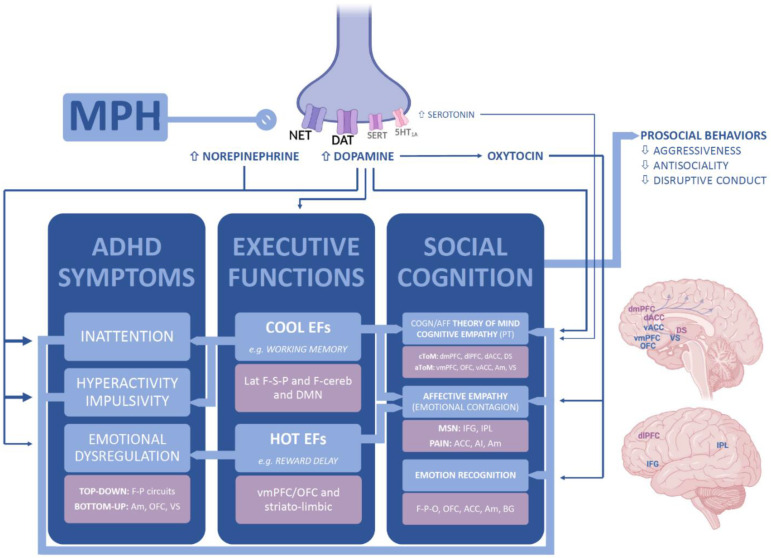
Proposed neurobiological pathways linking methylphenidate pharmacodynamic effects and changes in social cognition in patients with ADHD.

Based on our review, we could speculate that, in patients with ADHD, drug treatment improves social cognition skills, namely emotion recognition, empathy and ToM abilities. Psychostimulant treatment has been also likely associated with a long-term improvement in prosocial behavior and other outcomes of social functioning [72], including social judgment and interpersonal relationships [56]. Empathy is a critical facilitator of prosocial behavior and is disrupted in ADHD patients, as previously reported in Section 1; however, the beneficial impact of pharmacological interventions on social cognition and functioning is likely to result from their effects on brain circuitries known to be involved in ADHD, possibly, but speculatively, not exhaustively mediated by the effects on the core symptoms of the disorder. In the subsequent paragraphs, we try to illustrate a theoretical framework linking psychostimulants mechanisms of action to social cognition outcomes in ADHD that could be hypothesized based on literature findings, which is depicted in Figure 2.

Structural and functional neuroimaging studies have documented abnormalities in brain anatomy and function in individuals with ADHD [73,74,75]. Meta-analyses of magnetic resonance imaging (MRI) studies show smaller volumes in the ADHD brain, most consistently in the basal ganglia [74,76]. Functional abnormalities are reported by a meta-analysis of 55 task-based functional MRI (fMRI) studies [73], reporting that children with ADHD show a hypoactivation in the fronto-parietal and ventral attentional networks, involved in attention and goal-directed behaviors, and a hyperactivation in the sensorimotor network and default-mode network, involved in lower-level cognitive processes [77]. In high-functioning, drug-naive young adults with ADHD, resting-state fMRI revealed altered connectivity in the orbitofrontal-temporal-occipital and frontal-amygdala-occipital networks, relating to inattentive and hyperactive/impulsive symptoms, respectively, compared with matched controls [78]. Structural MRI studies on children and adolescents with ADHD demonstrated that chronic naturalistic stimulant treatment was associated with attenuation of ADHD-related brain structural abnormalities, the more consistent findings in frontal, striatal, cerebellar and corpus callosum regions [79]. In the review by Spencer et al. [79], analyzed fMRI studies revealed most consistent findings for striatum and anterior cingulate cortex. In the review by Faraone [78], the author reported that MPH treatment was associated with an increased activation of the parietal and prefrontal cortices and with an increased deactivation of the insula and posterior cingulate cortex during visual attention and working memory tasks. The same author indicated that MPH exposure altered connectivity strength across various cortical and subcortical networks.

Interestingly, it has been suggested that the efficacy of psychostimulants on the core symptoms of the disorder—i.e., inattention, hyperactivity, and impulsivity—is due to the increased central dopaminergic and noradrenergic activity in the brain regions that include the cortex and the striatum, regions involved in the regulation of attentional and behavioral outcomes [80]. ADHD patients have, indeed, deficits in higher-level cognitive functions necessary for mature adult goal-directed behaviors, that is executive functioning (EF), which are known to be mediated by later developing fronto-striato-parietal and fronto-cerebellar networks [81]. The most consistent deficits are in the so-called “cool” EF, such as motor response inhibition, working memory, sustained attention, response variability, and cognitive switching [81,82,83,84], as well as in temporal processing (i.e., motor timing, time estimation, and temporal foresight), with the most consistent deficits in time discrimination and estimation tasks [85,86]. However, impairment has also been found in so-called “hot” EF of motivation control and reward-related decision making, as measured in temporal discounting and gambling tasks, albeit with more inconsistent findings [82,86,87,88].

Among these, emotion regulation (ER) is also known to be affected in ADHD patients. According to what Posner and colleagues [89] termed the “dyscontrol hypothesis”, ER deficits—or simply emotional dysregulation (ED)—in ADHD arise from impairments in hot EF. ED is an altered ability to modulate emotional states in an adaptive and goal-oriented way, with excitability, ease anger, and mood lability [90], which should be considered as pivotal components of ADHD [91]. More specifically, deficits in top-down inhibitory processes, which are found in a sizeable portion of individuals with ADHD, would lead to abnormal emotional reactions, whilst emotional processing per se would be largely normal. The concept proposed by Barkley of “deficient emotional self-regulation” should be considered within this model [92]. Alternatively, the affectivity hypothesis posits that emotional processing per se is abnormal, due to dysfunctions in bottom-up circuits, encompassing the amygdala, the orbitofrontal cortex, and the ventral striatum that processes emotional stimuli.

Interestingly, previous studies demonstrated a positive correlation between EF/ER and empathy/ToM competences in healthy subjects (for a recent review on the topic, please refer to [52]); inhibitory control, working memory, and cognitive flexibility were more strongly related to cognitive empathy, while only inhibitory control was closely related to the affective component. Conversely, a recent paper by our research group [7], which assessed the reciprocal relationship between empathic attitudes and executive functioning in ADHD patients with comorbid conditions, indicated that this latter was more strongly related to the affective empathy. Moreover, a recent meta-analysis [93] that examined the effects of MPH on executive functions in children, youths, and adults with ADHD found that the effects on response inhibition, working memory, and sustained attention were small to moderate. Thus, one may speculate that MPH has a positive effect on EF that, in turn, constitutes a possible mediator for the improvement of empathy and social abilities in youths with ADHD.

From a neuroanatomical point of view, strong evidence supports a model of two separate, yet interacting, systems for empathy, as previously mentioned in Section 1. AE would rely on a large brain network that includes the anterior cingulate cortex, anterior insula, inferior parietal lobule, and inferior frontal gyrus, with its mirror neuron system involved in motor imitation and emotional contagion [14,94]. On the other hand, CE/ToM is subserved by prefrontal and temporal networks: the cognitive ToM network engages the dorsomedial prefrontal cortex, dorsal anterior cingulate cortex, and dorsal striatum; the affective ToM network engages the ventromedial prefrontal cortex, orbitofrontal cortices, ventral anterior cingulate cortex, amygdala, and ventral striatum [94]. A functionally interactive dorsal and ventral attention/selection system at the temporoparietal junction and anterior cingulate cortex modulates the ability to distinguish between self and other mental states [24]. From a neurochemical point of view, AE is modulated in part by oxytocinergic projections [33], while, on the other hand, CE/ToM functioning is dependent on the integrity of the dopaminergic and serotoninergic systems [24].

Pharmacological treatments, especially with MPH, through a direct modulation of central dopaminergic and noradrenergic transmission in cortex and striatum, an indirect action on other neuropeptides such as oxytocin, and by regulating neural activity in these systems acting on top-down and only partly on bottom-up circuits [90], can concurrently improve empathy, theory of mind, executive and emotional regulation in youths with ADHD (see Figure 2). On the other hand, we may speculate that a possible mechanism explaining the social effect of psychostimulants in ADHD youths may be a positive effect of improved attention and EF on empathic abilities.

Little literature evidence is available to discern whether the impacts of stimulants on social cognition could be mediated by the complementary effects on the different core symptoms of ADHD, namely inattention and hyperactivity/impulsivity. Indeed, the studies conducted so far have not specifically addressed the effects of MPH on different ADHD subtypes, but it may be argued that different subtypes could present different empathy profiles. Children with a predominant Inattentive subtype are typically less aggressive and less likely to have comorbid ODD/CD than children with the combined or the Hyperactive/Impulsive subtype that seem to be less empathic than youths with the inattentive one [46,58]. We could speculate that the Inattentive and Hyperactive/Impulsive profile lies intermediate between two extremes, the latter being substantially overlapping with that of ODD/CD. The very few fMRI studies that compared non-comorbid ADHD and ODD/CD children showed that ADHD is associated with dorsolateral prefrontal and inferior frontal under-activation, while ODD/CD was associated with paralimbic under-activation in orbitofrontal, limbic, and superior temporal regions [83,85,88].

Empathy deficits have been implicated in several neurodevelopmental disorders, among which ASD is the most studied [9,95]. Some authors have speculated that performances of individuals with ADHD on social cognition tasks lies intermediate between ASD and healthy controls [43]. Socioemotional problems in ADHD are associated with a more negative prognosis, notably interpersonal and educational problems and an increased risk of developing other psychiatric disorders, while on the other hand, attentional problems at a very early age have been supposed to precede the onset of clinical manifestations of ASD, ADHD, or both disorders [96]. In this perspective, the association between ASD and ADHD traits may be featured by shared attention-related problems (inattention and attentional switching capacity) and biological pathways involving attentional control may be a key factor in the overlapping conditions [97,98]. Future studies are welcome to explore the effects of MPH on empathy/ToM and emotion recognition also in patients with co-occurring ASD.

The current review indicates several limitations of the studies on this topic. First, the limited number of eligible studies. Second, the heterogeneity of the recruited samples and the study protocols (single dose of MPH versus mid-term treatment). Third, the use of self- and parent-rated measures of empathy, which should be integrated with experimental paradigms. In future investigations, empathy/ToM abilities and emotion recognition skills should be assessed in separate samples of ADHD patients including the Inattentive, Hyperactive/Impulsive, and Combined subtype carefully matched on age, gender and medication status. In addition, it would be interesting to investigate possible different responses on the bases of the comorbidity such as other neurodevelopmental disorders, specially ASD, or psychiatric comorbidity.

## 5. Conclusions

This review provides a contribution for a better understanding of the possible effects of the MPH. Some evidence support the notion that the timely and affective treatment of ADHD symptoms may have beneficial effects not only on core symptoms of ADHD, but also on the social difficulties of youths with ADHD. Future studies on the association of several measures of empathy with comorbid disorders, such as ASD and disruptive behavioral problems, are warranted. At the same time, future studies concerning gender effects are desirable. One important issue for future studies would be the question of whether empathy/Tom/emotion recognition impairments can be observed in all subtypes and, in this case, whether the underlying mechanisms are the same for ADHD subtypes.

## Figures and Tables

**Table 1:** Summary findings of the included studies: empathy and theory of mind.

Study	N	Gender	Age	ADHD	Comorbidity	Treatment	Assessment	Outcome
Coelho et al., 2017 [65]	60 ADHD (30 C, 30 I)	48/12	7–14 (unimodal group = 10.13) (multimodal group = 10.2)	no other medications when recruited	ID excluded	unimodal-medication only vs. multimodal medication + CBT for 20 weeks (prolonged release-MPH 20 mg)	Children’s Social Skills Multimedia System	Multimodal treatment showed more improvement in frequency indicators on empathy.
Demirci and Erdogan, 2016 [58]	60 ADHD (21 C, 17 H/I, 22 I) 60 HCs	35/25 ADHD 35/25 HCs	8–15 (ADHD = 10.8) (HCs = 10.8)	drug-naive	ID, ASD, CD excluded	pharmacological treatment for 12 weeks: −38 OROS-MPH (final dose 1.2 mg/kg/day) −32 ATX (final dose 1.2 mg/kg/day)	RMET	The ADHD sample had significantly lower scores in RMET than HCs. ADHD-H/I had a lower number of correct answers in the RMET than ADHD-I. After OROS-MPH/ATX treatment, the ADHD sample showed a significant improvement in RMET.
Fantozzi et al., 2021 [62]	61 ADHD (50 C, 11 I)	51/10	6–17 (10.3)	drug-naive	ID, ASD excluded 14 SLD; 9 ODD; 4 MD; 2 LD; 1 AD; 1 tics; 1 dyspraxia	MPH treatment for 6 months (final dosage 31.6 ± 15.1 mg/day)	BES	Significant improvement in AE and CE. Changes in attention symptoms predicted changes in AE but not in CE.
Golubchik and Weizman, 2017 [59]	52 ADHD		8–18	psychostimulant-medication naive	ID, ASD, schizophrenia, bipolar disorder, suicidal ideation excluded 26 ODD	MPH treatment for 12 weeks (0.5–1 mg/kg/day)	EQ-C	Significant improvement in EQ scores in both groups (ADHD and ADHD/ODD). Only in the ADHD group, a significant correlation between changes in ADHD-RS and in EQ-C was found.
Golubchik and Weizman, 2019 [66]	25 ADHD	21/4	7–17 (10.8)		ID, ASD, psychosis, bipolar disorder excluded	single dose of MPH (1 mg/kg)	RMET	No improvement of RMET.
Gumustas et al., 2017 [60]	65 ADHD 61 HCs	53/12 ADHD 46/15 HCs	8–14 (ADHD = 10.86) (HCs = 11.21)	drug-naive	ID, ASD, psychosis, mood disorders, anxiety disorders, ODD excluded	OROS-MPH treatment for 12 weeks (0.83 ± 0.21 mg/kg/day)	BEI (trait empathy) GEM-PR (trait empathy) ERT (state empathy)	No significant statistical differences in trait and in state empathy skills in the two groups. Following the MPH treatment, the ADHD group showed a significant increase in the ERT (state empathy) interpretation sub-score.
Levi-Shachar et al., 2019 [61]	50 ADHD 40 HCs	28/22 ADHD 22/18 HCs	6–12 (ADHD = 9.42) (HCs = 8.95)	psychotropic medication free	psychosis, affective disorders, CD, substance abuse disorder excluded	single dose of short-acting MPH (0.3–0.5 mg/kg)	ToM test	The ADHD sample displayed significantly poorer ToM performance compared with HCs. Following MPH administration, the ToM performance of the ADHD sample normalized.
Levi-Shachar et al., 2021 [67]	50 ADHD	28/22 ADHD	6–12 (ADHD = 9.42)	psychotropic medication free	psychosis, affective disorders, CD, substance abuse disorder excluded	single dose of short-acting MPH (0.3–0.5 mg/kg)	ToM test FPR	Negative association between severity of behavioral ADHD domains and impairment in ToM. Administration of MPH improved ToM performance, with the greatest improvement in children with more severe behavioral symptoms.
Maoz et al., 2013 [47]	24 ADHD (11 C, 13 I)	16/8	6–12 (10.2)		ID, psychosis, bipolar disorder, major depression, DBD, substance abuse disorder excluded	single-dose of long-acting MPH	IRI FRP TCT	Significant improvement in ToM performance.
Maoz et al., 2019 [46]	24 ADHD 36 HCs	6/8 ADHD 19/17 HCs	6–12 (ADHD = 10.29) (HCs = 9.37)	psychotropic medication free	ID, psychosis, bipolar disorder, major depression, CD, substance abuse disorder excluded	single dose of long-acting MPH	IRIFRP	The ADHD sample showed lower levels of self-reported empathy and FRP scores compared with HCs. In ADHD sample, MPH administration improved FRP scores to a level equal to that in HCs.

Abbreviations: AE, Affective Empathy; ADHD, Attention Deficit/Hyperactivity Disorder; ADHD-RS, Attention Deficit/Hyperactivity Disorder-Rating Scale; ASD, Autism Spectrum Disorder; ATX, Atomoxetine; BEI, Bryant Bryant Index of Empathy; BES, Basic Empathy Scale; C, Attention Deficit/Hyperactivity Disorder-Combined subtype; CE, Cognitive Empathy; CD, Conduct Disorder; EQ-C, Empathizing Quotient-Children’s version; ERT, Empathy Response Task; FPR, Faux-Pas Recognition task; GEM-PR, Griffith Empathy Measurement-Parent Rating; H/I, Attention Deficit/Hyperactivity Disorder-Hyperactive/Impulsive subtype; HCs, Healthy Controls; I, Attention Deficit/Hyperactivity Disorder-Inattentive subtype; ID, Intellectual Disability; IRI, Interpersonal Reactivity Index; RMET, Reading the Mind in the Eyes Test; MPH, Methylphenidate; ODD, Oppositional Defiant Disorder; OROS-MPH, long acting-Methylphenidate; SLD, Specific Learning Disability; TCT, ToM Computerized Task; ToM, Theory of Mind.

**Table 2:** Summary details of the included studies: Emotion Recognition.

Study	N	Gender	Age	ADHD	Comorbidity	Treatment	Assessment	Outcome
Demrici and Erdogan, 2016 [58]	60 ADHD (21 C, 17 H/I, 22 I) 60 HCs	35/25 ADHD 35/25 HCs	8–15 years (ADHD = 10.8) (HCs = 10.8)	drug-naive	ID, ASD, CD excluded	pharmacological treatment for 12 weeks: −38 OROS-MPH (final dose 1.2 mg/kg/day) −32 ATX (final dose 1.2 mg/kg/day)	BFRT	ADHD sample had significantly lower scores in BFRT than HCs. ADHD-H/I had a lower number of correct answers in BRFT than ADHD-C and I. After OROS-MPH/ATX treatment, the ADHD sample showed a significant improvement in BFRT.
Gumustas et al., 2017a [60]	65 ADHD 61 HCs	53/12 ADHD 46/15 HCs	8–14 years (ADHD = 10.86)(HCs = 11.21)	drug-naive	ID, ASD, psychosis, mood disorders, anxiety disorders, ODD excluded	OROS-MPH treatment for 12 weeks (0.83 ± 0.21 mg/kg/day)	DANVA-2	No significant statistical differences in facial expression recognition skills in the two groups. Following the MPH treatment, the ADHD group showed a significant decrease in the recognition error of anger and sadness expressions.
Hall et al., 1999 [68]	15 ADHD (13 C, 2 H/I) 15 ADHD/LD (14 C, 1 H/I) 15 no ADHD or LD	36/9	7–10 years	the ADHD sample was taken MPH (Ritalin) for at least a month at the time of the study	ID excluded	the DANVA was administered twice to each child in the ADHD and ADHD/LD groups: once while the ADHD and ADHD/LD participants were on medication and once off medication	DANVA SPBRS	The ADHD/LD group demonstrated significant difficulty in comparison to their peers in perceiving paralanguage cues effectively. The ADHD/LD group showed significant improvement on the Postures and Paralanguage subtests during on-medication conditions.
Schulz et al., 2018 [69]	25 ADHD (17C, 8I)	14/9	19–52 years (34.8 ± 9.8)	2 participants were on medication at intake, 9 had a history of previous stimulant treatment (2 of whom had also previously been treated with nonstimulant medication)	psychosis, BD, PTSD, substance use disorderexcluded	3 to 4 weeks of LDX (mean maintenance dose = 64 mg/day–SD = 13 mg) treatment and 3 weeks of medication in a randomized, counterbalanced, hybrid crossover design	participants were scanned twice with event-related fMRI while performing an emotional go/no-go task	No significant differences between the two treatment arms. LDX was associated with an increase in fMRI activation in the right amygdala and reduced interactions with the orbital aspect of the left inferior frontal gyrus specifically for responses to sad faces.
Schwenck et al., 2013 [70]	56 ADHD (10C,2H/I,44I) 28 ADHD-MD− 28 ADHD-MD+ 28 CG	19/9	8.2–17.3 years (MD− = 12.36) (MD+ = 12.31) (CG = 12.49)	47 children in the ADHD group were taken MPH at the time of the study (one child was additionally taken ATX), 6 drug-naive	ID, ASD, ODD, CD excluded	cross-sectional design study	MT	No differences found between ADHD-MD−, ADHD-MD+ and CG on emotion recognition.

Abbreviations: ADHD, Attention Deficit/Hyperactivity Disorder; ADHD-MED-, Attention Deficit/Hyperactivity Disorder no medication; ADHD-MED+, Attention Deficit/Hyperactivity Disorder with medication; ASD, Autism Spectrum Disorder; ATX, Atomoxetine; BD, Bipolar Disorder; BRFT, Breton Face Recognition Test; C, Attention Deficit/Hyperactivity Disorder-Combined subtype; CD, Conduct Disorder; CG, control group; DANVA, Diagnostic Analysis of Nonverbal Accuracy; ERP, event related potential; FEFA, Frankfurt Test and Training of Facial Affect; fMRI, functional magnetic resonance imaging; H/I, Attention Deficit/Hyperactivity Disorder-Hyperactive/Impulsive subtype; HCs, Healthy Controls; I, Attention Deficit/Hyperactivity Disorder-Inattentive subtype; ID, Intellectual Disability; LD, Learning Disability; LDX, lisdexamfetamine; MPH, Methylphenidate; MT, Morphing Task; ODD, Oppositional Defiant Disorder; OROS-MPH, long acting-Methylphenidate; PTSD, Post Traumatic Stress Disorder; SD, standard deviation; SPBRS, Social Perception Behavior Rating Scale.

## Data Availability

The data presented in this study are available on request from the corresponding author.

## Supplementary Materials

The following are available online at https://www.mdpi.com/article/10.3390/brainsci11111399/s1, Table S1: PRISMA checklist, Table S2: search strategy.
