# Supplementary material for: Biological Bases of Empathy and Social Cognition in Patients with Attention-Deficit/Hyperactivity Disorder: A Focus on Treatment with Psychostimulants
Source: Brain Sci. 2021 Oct 24;11(11):1399. doi: 10.3390/brainsci11111399 (PMC8615705; doi:10.3390/brainsci11111399)
Supplement: Supplementary file 1 [file brainsci-11-01399-s001.zip › Supplementary Table S1.pdf]

PRISMA 2009 Checklist

Section/topic	#	Checklist item	Reported on page #
**TITLE**			
Title	1	Biological bases of empathy and social cognition in patients with Attention-Deficit/Hyperactivity Disorder: a focus on treatment with psychostimulants.	1
**ABSTRACT**			
Structured summary	2	Background: In recent years, there is growing interest in investigating the effect of specific pharmacological treatments for ADHD not only on its core symptoms, but also on social skills in youths. This stands especially true for ADHD patients displaying impulsive aggressiveness and antisocial behaviors, being the comorbidity with Disruptive Behavior Disorders one of the most frequently observed in clinical settings. Aim: This systematic review aimed to synthesize research findings on this topic following PRISMA guidelines and to identify gaps in current knowledge, future directions and treatment implications. Methods: Search strategies included the following terms: ADHD; Methylphenidate and other ADHD drugs; Empathy, Theory of Mind and Emotion Recognition. Full-text articles were retrieved and data from individual studies were collected. Results: Thirteen studies were finally included in our systematic review. Eight studies assessing changes in Empathy and/or Theory of Mind in patients with ADHD treated after pharmacological interventions were identified. Similarly, seven partially overlapping studies assessing changes in Emotion Recognition were retrieved. Conclusion: Despite a great heterogeneity in methodological characteristics of the included studies, most of them reported an improvement in emphatic and theory of mind abilities in youths with ADHD treated with psychostimulants and nonstimulant drugs. Positive but less consistence results about emotion recognition performances.	1
**INTRODUCTION**			
Rationale	3	Recent literature suggests that pharmacological interventions in ADHD patients may provide beneficial effects on social cognition deficits. Indeed, psychostimulants, including methylphenidate (MPH) and amphetamines, the gold-standard drug treatment for ADHD, have been likely associated with improvements in social judgment and interpersonal relationships, as well as in empathy and ToM in youths with ADHD. Interestingly, MPH administration has been shown to promote empathy-like behaviors and sociability and reduce aggressiveness in a mouse model of callousness. Moreover, it has been suggested that MPH treatment may possibly provide an improvement in emotion recognition. Nonetheless, the evidence on the efficacy of psychostimulants on empathy and ToM, as well as on emotion recognition, is still under debate.	4
Objectives	4	The aim of the present study was to systematically review the available literature on the topic in order to clarify whether the gold-standard drug treatment for ADHD may exert its effects on empathy and related constructs, through and beyond its well-known effects on the core symptoms of the disorder.	4
**METHODS**			
Protocol and registration	5	The protocol of the present systematic review was preregistered on PROSPERO (CRD42021247024).	4
Eligibility criteria	6	Study design: any type of clinical trial; Comparison: either case versus control, drug versus placebo or pre- to pe-ri-/post-treatment; Participants: patients non-retrospectively diagnosed with ADHD according to the international classification systems DSM-IV, ICD-9 or later versions; no restriction for participants' age, gender or IQ; Intervention: either one-day single-dose administration or prolonged daily administration of psychostimulants (e.g.	5

		Methylphenidate) or nonstimulant drugs (e.g. Atomoxetine); Measures: any type of measurement (i.e. tasks, rating scales and parent- or self-rated questionnaires) assessing empathy, theory of mind and emotion recognition.	
Information sources	7	Three bibliographic databases were searched, namely PubMed, Scopus and Web of Science, from the inception date to the 10th August 2021.	4
Search	8	A search strategy was developed including three groups of terms related to the following sematic fields: (1) ADHD; (2) Methylphenidate or other psychostimulants and nonstimulant drugs for ADHD; (3) Social Cognition, Empathy, Theory of Mind and Emotion Recognition.	4
Study selection	9	Results of the bibliographic search were then downloaded into Mendeley software and two authors (Gianluca Sesso and Pamela Fantozzi) reviewed and discussed the scoping search which included both original studies and reviews. If a previous review was already available on the topic, its reference list was carefully searched to retrieve primary studies. Reference lists of the studies included in the final search were also thoroughly inspected to identify relevant citations.	4
Data collection process	10	Our search strategy was used to retrieved potentially relevant abstracts; duplicates from different bibliographic databases were initially removed, whereas additional records identified through reference lists inspection of screened articles, as stated above, were also included. Two researchers (Gianluca Sesso and Pamela Fantozzi) screened all titles and abstracts to identify relevant articles. Full texts of selected papers were then retrieved and carefully screened to finally identify included studies according to eligibility criteria. Any disagreements were resolved by consensus.	5
Data items	11	For each included study, we extracted relevant information, whenever available, including sample size, demographic data (age and gender), ADHD diagnosis and subtypes, intellectual functioning and psychiatric comorbidities, previous and current medication (including dosage and administration), follow-up duration, as well as information about the clinical measure used to assess changes in empathic competencies and related con-structs and main findings of the study. When datasets were not fully available, authors of the included studies were contacted to attain the relevant data.	6
Summary measures	12	Summary outcomes were descriptively reported in the narrative synthesis.	–
Synthesis of results	13	Methods of handling data and combining results of studies were not applied.	–
**RESULTS**			
Study selection	14	In summary, 1193 abstracts were initially retrieved using our search strategy, plus one additional record identified in the reference lists of the studies included in the final search. After duplicates removal, 724 records were screened by two authors (G.S. and P.F.) and any disagreement was resolved by consensus. Twenty-seven full-text articles were carefully assessed for eligibility, of which 12 were excluded. Fifteen articles were finally included in our systematic review and non-mutually subdivided into two partially overlapping groups as follows: (1) empathy and theory of mind (n = 10 studies); (2) emo-tion recognition (n = 7 studies).	9
Study characteristics	15	For each study, extracted information are available in Tables 1 – 2.	6
Results of individual studies	16	For each study, extracted information are available in Tables 1 – 2.	6

**DISCUSSION**			
Summary of evidence	17	The present systematic review aimed to synthesize research findings on the effect of psychostimulants and nonstimulant drugs on social cognition in patients with ADHD. As far as we know, our study is the first review that systematically and specifically addressed this topic; former narrative but still comprehensive reviews were respectively focused on social dysfunctions in ADHD, with the contribution of comorbid disruptive behavior disorders (i.e. ODD/CD) to social impairments, and on the link between social cognition deficits in ADHD and evidence from neuroimaging and lesion studies. Here, we complementarily aimed at looking for the available evidence from scientific literature on the impact of pharmacological interventions on empathy, theory of mind and emotion recognition in ADHD.	11
Limitations	18	The current review indicates several limitations of the studies on this topic. First, the heterogeneity of the recruited samples and the study protocols (single dose of MPH versus mid-term treatment). Second, the use of self- and parent-rated measures of empathy, which should be integrated with experimental paradigms. In future investigations, empathy/ToM abilities and emotion recognition skills should be assessed in separate samples of ADHD patients including the Inattentive, Hyperactive/Impulsive and Combined subtype carefully matched on age, gender and medication status. In addition, it would be interesting investigating possible different responses on the bases of the comorbidity such as other neurodevelopmental disorders, specially ASD, or psychiatric comorbidity.	15
Conclusions	19	This review provides a contribution for a better understanding of the possible effects of the MPH. Some evidences support the notion that the timely and affective treatment of ADHD symptoms may have beneficial effects not only on core symptoms of ADHD, but also on the social difficulties of youths with ADHD. Future studies on the association of several measures of empathy with comorbid disorders, such as ASD and disruptive behavioral problems, are warranted. At the same time, future studies concerning gender effects are desirable. One important issue for future studies would be the question whether empathy/Tom/emotion recognition impairments can be observed in all subtypes and, in this case, whether the underlying mechanisms are the same for ADHD subtypes.	15
**FUNDING**			
Funding	20	This research received no external funding.	15
